# Production of Aldehydes by Biocatalysis

**DOI:** 10.3390/ijms22094949

**Published:** 2021-05-06

**Authors:** Veronika Kazimírová, Martin Rebroš

**Affiliations:** Institute of Biotechnology, Faculty of Chemical and Food Technology, Slovak University of Technology, 811 07 Bratislava, Slovakia; veronika.kazimirova@stuba.sk

**Keywords:** biomaterials, gene expression/regulation, green chemistry, metabolism, natural products, protein engineering

## Abstract

The production of aldehydes, highly reactive and toxic chemicals, brings specific challenges to biocatalytic processes. Absence of natural accumulation of aldehydes in microorganisms has led to a combination of in vitro and in vivo strategies for both, bulk and fine production. Advances in genetic and metabolic engineering and implementation of computational techniques led to the production of various enzymes with special requirements. Cofactor synthesis, post-translational modifications and structure engineering are applied to prepare active enzymes for one-step or cascade reactions. This review presents the highlights in biocatalytical production of aldehydes with the potential to shape future industrial applications.

## 1. Introduction

The biocatalytic advances of aldehyde synthesis, the selection of aldehyde-producing enzymes and aldehyde-forming natural cascades have been a research focus for decades. The great diversity in the origin, catalytic function and products’ high reactivity is especially challenging in their production [1,2]. Modern methods of genetic and metabolic engineering allow the construction of novel routes for synthesis, even for products that are not present in nature. The combination of strategies based on in vitro and in vivo production results in a high variety of otherwise difficult-to-achieve products. Aldehydes are highly reactive and are, therefore, a toxic class of chemicals [1]. Despite their well-documented effects on cells, the mechanism of toxicity is understood only in general; for example, through the formation of covalent adducts with nucleophilic residues of macromolecules. This is mainly caused by the structural diversity of aldehydes and different targeted molecules at the same time [3].

Even if progress in the field of aldehyde metabolic production and its bioaccumulation notably increased, the toxicity of aldehydes would still hold the attention of many researchers. One strategy is to keep the concentration of toxic chemicals low (by in situ product removal, or by low substrate concentrations) to keep the biocatalyst unaffected by produced aldehydes [1,2,4].

Due to their toxicity, most natural microorganisms do not accumulate aldehydes [1]. Hence, they have become important intermediates in in vitro and in vivo enzymatic cascades for preparing highly reactive, short-living intermediates that demand only relatively simple conversion to a final product. The aldehyde intermediate determines the final complex chemical structure (e.g., various-length-chained aliphatic aldehydes, branch-chained aldehydes, aromatic aldehydes). The toxic effect can be minimized by making use of the “hidden reservoir” of aldehyde species [5] or the design of cascades so that aldehyde is produced in situ [6].

Aldehydes are predetermined for many industrial uses, such as value-added building blocks for the synthesis of large molecules, such as rubber and plastic [7]. They are mostly known for possessing distinctive organoleptic features and adding flavors such as nut, fat, fruit or grass to a final product. One of the most important flavor molecules—vanillin—is also an aromatic aldehyde [8]. This makes aldehydes a group of highly important chemicals applied in the food and fragrance industry [9,10]. These industries are important because of the increased ratio of health-conscious consumers to the demand of products that can be labelled as natural [11].

In this review, recent advances in the biocatalytic production of aldehydes are summarized. The presented enzymes catalyze a selection of biocatalytical processes that are, or possess the potential to be, industrially applicable. The use of whole-cell biocatalysts and approaches for aldehyde synthesis, including bioconversion and de novo synthesis by engineered microorganisms, are discussed. Finally, specific problems resulting from recombinant production of enzymes and analytical methods for the evaluation of aldehydes are summarized.

## 2. One-Step Bioconversion—Enzymes That Produce Aldehydes

Modern biocatalysis is a sophisticated method of synthesis of both bulk and fine chemicals. Novel-developed bio-based catalysts are used in environmentally friendly conditions and are an alternative to conventional catalysts (e.g., Ru, Fe [12]) [13]. By the application of the right enzyme, pure compounds can be obtained in one-step transformations. In comparison, chemical synthesis also exhibits many other drawbacks, such as poor selectivity and low yield, and generates environmental waste. The demand for products labelled as “natural” contributed to the application of enzyme-catalyzed reactions. The substrate specificity, regio- and enantio-selectivity and ability to operate under mild conditions led to the creation of an alternative for the production of fine chemicals by biocatalysis [9].

### 2.1. Aldehydes Produced as Primary Product

#### 2.1.1. Carboxylic Acid Reductases

Carboxylic acid reductases (CARs, EC 1.2.1.30) (or aldehyde oxidoreductases) catalyze the reduction of carboxylic acids to the corresponding aldehydes. CARs are large, structurally complex enzymes that usually demand additional cosubstrates and cofactors and post-translational modification to be active [14,15].

Aldehyde synthesis by the reduction of carboxylic acids gained significance after the successful expression of unique CARs from *Nocardia* sp. The enzymatic reduction of carboxylic acids at that time was an unexploited field that brought new potential routes for organic synthesis. Even though various organisms (from actinomycetes to white rot fungi) were tested for the reduction of carboxylic acids to their corresponding aldehydes, CARs posed unique catalytic activity that made them superior to classical chemical and enzymatic reduction paths [16].

**Figure 1:**
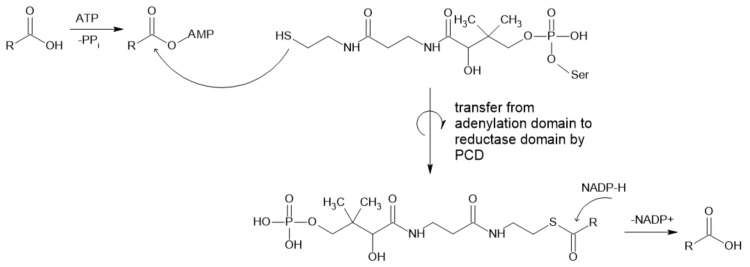
Mechanism of reaction catalyzed by CAR (PCD—peptidyl carrier domain).

CARs usually catalyze the reduction of straight chain (C2–C18), branched (C2–C8) [1] and aromatic carboxylic acids [15] to the corresponding aldehydes (Figure 1). The substrate specificity varies with enzymes obtained from different sources, while molecules with carboxylic acid as the only charged/polar functional group are preferred [17]. They can act on a broad range of substrates, from aliphatic to aromatic and polycyclic and heterocyclic acids [2], including benzoic, vanillic and ferulic acid [16]. The physiological function of these enzymes is not yet known, but it is possible that they play an unknown role in the non-standard metabolism of lipids [17]. CARs are nicotinamide adenine dinucleotide phosphate (NADPH)- and adenosine triphosphate (ATP)-dependent while forming adenosine monophosphate (AMP), pyrophosphate (PP_i_) and NADP^+^. Mg^2+^ is needed in in vitro applications as the counterion for ATP [18], which is required for activation of thermodynamically stable carboxylic acid prior to reduction to the aldehyde [1].

Their structure is complex, since proteins are relatively large (130 kDa [19,20]. Enzymes consist of substrate-activating N-terminal adenylation domains linked by a peptidyl carrier domain (PCD) to a C-terminal thioester reductase termination domain [20,21]. Carboxylic acid is the first adenylated domain, forming an acyl adenylate intermediate, while the final aldehyde product is formed by reduction with NADPH [19]. This final step depends on the docking of the 4-phosphopantetheine (Ppant) group at the active site of the enzyme, which leads to the reorientation of bound NADPH from a noncatalytic to a catalytically competent position. As proposed by Galoth et al. (2017), this change does not allow the reduction to proceed beyond aldehyde formation because this process is strictly a two-electron transfer. They also showed that the mutation of a single Asp residue involved in the reorientation of nicotinamide leads to the formation of alcohol by four-electron reduction [20].

CARs can be sourced from native producents (e.g., *Neurospora crassa*, *Nocardia iowensis*, *Mycobacterium marinum*) and in some cases used as a whole-cell biocatalyst [2]. However, recombinant production, which simplifies the cultivation and purification of CARs, brings new challenges, resulting from the need for post-translational modifications. Expression in *Escherichia coli* (*E. coli*) led to the formation of apoenzyme, which needs to be modified with the *Nocardia* phosphopantetheine transferase (PPTase) to form a holoenzyme. Co-expression of these two enzymes led to a 20-fold increase in CAR specific activity [19]. Cofactor issues are still a considerable obstacle that researchers must be aware of.

#### 2.1.2. Diamine Oxidase

**Figure 2:**
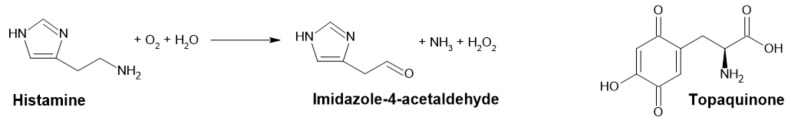
Reaction catalyzed by DAO and the structure of its cofactor topaquinone.

Diamine oxidase (DAO, EC 1.4.3.22), formerly known as histaminase, catalyzes the oxidative deamination of amines to their corresponding aldehydes, ammonia and hydrogen peroxide (Figure 2). The reaction proceeds through a two-stage ping-pong mechanism, while the preferred substrates include polyamines, such as putrescine, cadaverine and spermidine, even though it is best known for the degradation of histamine [22,23,24,25].

DAO belongs to a family of copper-containing amine oxidases (AOs), which have a typical homodimeric structure. Every active site contains one Cu^2+^ ion and topaquinone cofactor. Moreover, each subunit contains two additional binding sites for cations (presumably Ca^2+^) with unknown function. The size of DAO is 140–200 kDa [22,23] and contains a highly conserved aspartate residue (D186 in human DAO (hDAO) [26]). This is very specific for all DAOs but is not present in other AOs. It is responsible for the specificity to diamines, while it mediates the H-bond with the second amino group of diamine substrates. This interaction is vital for substrate interaction and positioning in active site cavity [22,26].

hDAO is in the center of attention because of the presumption that it mediates extracellular degradation of histamine [24], which is the main mediator of allergic reactions [27]. The hDAO concentration rises several hundredfold and reaches a maximum concentration in the 20th week of gestation [24]. Insufficient hDAO production can lead to intolerance of histamine containing foods. This is why it is widely used as a food supplement to lower the histamine content in the gastrointestinal tract [25]. Despite extensive research, the role of hDAO in histamine pathogenesis remains unknown [23].

Recombinant production of hDAO was accomplished for the first time in 2002 [28]. In this case, insect cells as hosts were used and characterization of the enzyme was carried out. However, insect cells produced non-human-like glycosylation, which can affect enzyme properties. In 2016, Gludovacz et al. prepared recombinant hDAO using chines hamster ovary cells. After removal of the cells, the supernatant contained 0.02 g L^−1^ hDAO. This type of production enabled researchers to address important topics, such as screening of chemical libraries for DAO inhibitors, production of monoclonal antibodies for reliable enzyme linked immunoassays (ELISA) for precise DAO quantification in biological samples and recombinant hDAO use for rapid histamine degradation during anaphylaxis [23].

#### 2.1.3. Alcohol Oxidase

**Figure 3:**
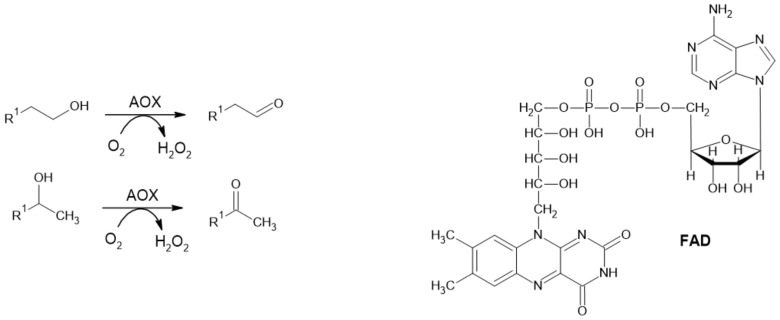
Reaction catalyzed by AOX and its cofactor FAD.

Alcohol oxidase (alcohol: O_2_ oxidoreductase; EC 1.1.3.13; AOX) catalyzes the irreversible oxidation of primary and secondary alcohols to their corresponding aldehydes and ketones, respectively (Figure 3). Molecular oxygen serves as a terminal acceptor of electrons while releasing hydrogen peroxide as a by-product. Structurally, AOXs consist of multiple subunits, and each of them contains strongly bound flavine adenine dinucleotide (FAD) as a prosthetic group [29,30,31].

Typically, AOX occurs in methylotrophic yeast of *Pichia, Candida, Ogateae* and *Komagataella* spp. Its natural function is to mediate the conversion of methanol as a carbon source to formaldehyde, which is further metabolized by yeast katabolic pathways [29]. In brown-rot fungi, AOX is involved in the process of plant biomass degradation. It starts with the degradation of lignin, mediated by O-demethylases and other lignolytic enzymes. Hydrogen peroxide that is generated by AOX from methanol assists in other chemical modifications of cellulose, hemicellulose and lignin [31].

An interesting example is AOX from Gram-negative *Ochrobactrum* sp. AIU 033 recombinantly prepared in *E. coli*. The structure of this AOX differs from typical AOXs, while it consists of α2β2 hetero subunit structure (α subunit—54 kDa, β subunit—14 kDa). This enzyme showed catalytic activity toward primary alcohols (C_2_–C_10_) and glycolic acid. In the N-terminal region of the β subunit, a twin-arginine translocation (TAT) sequence was recognized and an enzyme was expressed in the periplasm of *E. coli*. Co-expression with *tatABC* increased the productivity of this enzyme [32].

One of many interesting products of AOXs is benzaldehyde, produced by the oxidation of benzyl alcohol. It can also be used for flavor enhancement of various natural products; for example, the treatment of orange water-phase essence [30]. Another interesting use of AOX is in biosensors for the analysis of alcohol and formaldehyde content in food articles. In this case, hydrogen peroxide is measured analytically [29,30]. The substrate tolerance and applications of AOXs were discussed in a recent review [12].

### 2.2. Aldehydes Produced as Intermediates

#### 2.2.1. Alcohol Dehydrogenase

**Figure 4:**
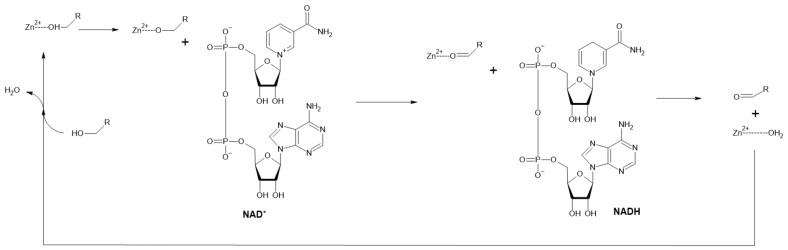
Reaction mechanism of ADH with Zn^2+^ is bound in its active site (Adapted with permission from ref. [35]. Copyright 2019 Academic Press).

Alcohol dehydrogenases (ADHs) catalyze the cofactor-dependent oxidation of alcohols to corresponding aldehydes or ketones. They can be classified based on cofactor requirements to NAD(P)-dependent, pyrroloquinoline quinone, heme, F420 (8-hydroxy-5-deazaflavin) or FAD-dependent enzymes. Moreover, NAD-dependent ADHs are subdivided into three types based on the alcohols metabolized to AHD type I (short-chain), ADH type II or Zn-ADH (medium-chain) (mechanism displayed in Figure 4) and ADH type III or Fe-ADH (long-chain) [33]. They are predominantly used for enantioselective syntheses of various chiral compounds [34] but their ability to produce aldehydes was also exploited.

The discovery of ADHs allowed for the rise of many applications in various fields, such as the pharmacological industry. At the same time, this resulted in extensive study of these enzymes for the purpose of improvement of their properties (e.g., stability, catalytical activity, stereoselectivity, substrates specificity) [36,37]. The most important recent accomplishment is probably the introduction of the “switches” from NADP^+^ to NAD^+^ and vice versa. For example, using semi-rational design to engineer *Thermoanaerobacter ethanolicus* ADH to change the selectivity from NADPH towards NADH [38]; for the reverse, the structure-based engineering of NAD^+^-dependent long-chain secondary ADH to accept NADP^+^. The latter was also coupled to redox-neutral biotransformations of C18 [39]. This is especially important for designing cascade processes and cofactor-coupled planning in the design of multi-enzymatic systems. Advances in this field were covered in a recent review [40].

ADHs are mostly used for biocatalysis and in vitro and in vivo cascades. Their applications for intermediate synthesis are significant. The construction of combinable enzyme modules, which include non-canonical ADH from *Pseudomonas putida*, (*P. putida*), can form chiral α-hydroxy acids, 1,2-amino alcohols and α-amino acids from terminal alkenes [41]. The combination of ADHs with transaminases (ω-TA) can be used for production on various non-natural (di)amines. A three-enzyme-cascade, consisting of *Bacillus stearothermophilus* ADH, *Vibrio fluvialis* ω-TA and L-alanine dehydrogenase from *Bacillus subtillis* expressed in *E. coli,* was used for the construction of effective whole-cell biocatalyst. This allowed the regeneration of cofactors (NAD^+^ for ADH and pyridoxal-phosphate for ω-TA) and various primary aliphatic, mono- and di-alcohols and aromatic alcohols were produced with the addition of only L-alanine and NH_4_Cl to the reaction buffer with resting cells [42].

In 2017, Bayer et al. reported the co-expression of ADH from *P. putida* in combination with CAR from *Nocardia iowensis* (with supporting enzyme PPTase) to adjust the redox equilibrium between alcohol, aldehyde and carboxylic acid species and keep the concentration of toxic aldehydes below levels that can destroy the cell. This allowed the synthesis of aldol product (*(3S,4R)*-1,3,4-trihydroxy-5-phenylpentan-2-one) from “hidden aldehyde pool” by another co-expressed enzyme—dihydroxyacetone-dependent aldolase from *E. coli* in 70% yield [5].

#### 2.2.2. α-Dioxygenase

**Figure 5:**
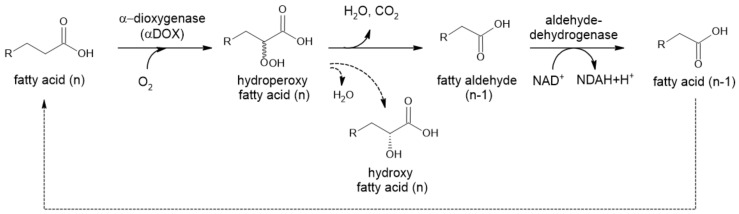
α-Oxidation in plants. Chemical decomposition of hydroperoxyl fatty acid to CO_2_ and aldehyde is caused by intramolecular mechanism. When reduction takes place, only D-isomer is detected (Adapted with permission from ref. [45]. Copyright 2014 Elsevier).

α-Dioxygenases (α-DOX) are monomeric heme-containing enzymes responsible for the first step of α-oxidation in plants (Figure 5). They oxidize medium-chain C_n_ fatty acids to 2-hydroperoxy fatty acids, which are spontaneously decarboxylated to form C_n−1_ fatty aldehydes [43,44]. These compounds (C_8_–C_13_) are commonly used as fragrance and aroma compounds with fresh, citrus and waxy odors. Plant α-dioxygenases require molecular oxygen as a co-substrate. Most of these enzymes also possess peroxidase activity, forming α-hydroxy fatty acid, except for rice DOX.

An *Oryza sativa* DOX with no peroxidase activity was recently expressed in *E. coli* BL21(DE3). Resting cells were successfully used to convert hexadecanoic, tetradecanoic, dodecanoic and decanoic fatty acids to form corresponding aldehydes and were easily recycled. This process was reported to be superior to other natural processes for fatty aldehyde synthesis, based on its high productivity (up to 1 g L^−1^ h^−1^ of pentadecanal, product yield of approx. 90%). The author also compared this approach to the application of CARs; while they require ATP, they also need an extensive reaction time (up to 24 h) and show low product yields (<10%) [43].

α-DOX has applications in the construction of in vivo biosynthetic routes. One of the most important examples is microbial synthesis of odd-chain fatty alcohols in *E. coli*, as the natural profile of products derived from fatty acid biosynthesis is fixedly even-chained. While this is the case when aldehydes stand as intermediates, it is clear that the application of this enzyme promises a new strategy in the biosynthesis of alcohols and alkanes [43,44].

These applications, however, reveal a problem linked to the design of biocatalytic α-DOX processes. Mediation of substrates, which can be solid under operational conditions, have low water solubility and cell membrane transport limitations need to be considered. This is often achieved by the treatment of cells with various detergents, such as Triton X-100. However, this caused leakage of the expressed enzyme and cell lysis, which limits its application for repeated use [43]. A more sophisticated approach for whole-cell biocatalysis is the overexpression of fatty acid transporter (FadL or its variant), which allows hydrophobic substrates to reach cytoplasm [46,47].

#### 2.2.3. Pyruvate Decarboxylase

**Figure 6:**
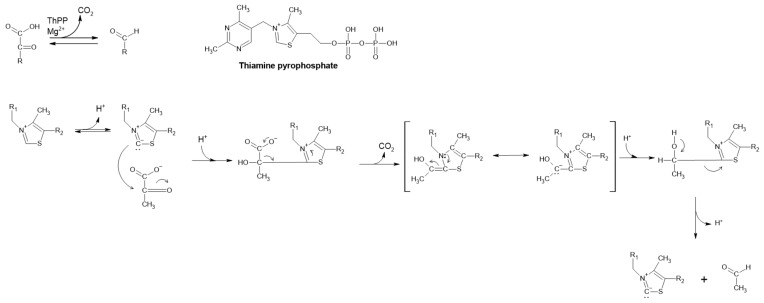
Reaction mechanism of PDC and its cofactor, ThPP.

The natural function of pyruvate decarboxylase (PDC) is to convert pyruvate to acetaldehyde via non-oxidative decarboxylation of α-keto acids and transfer of the acyl group (Figure 6) [1,48]. It is the key enzyme in all homo-fermentative ethanol pathways [49]. This process is Mg^2+^ dependent and requires the metabolically active coenzyme thiamine pyrophosphate (ThPP). Interestingly, ThPP catalyzes this reaction alone but with lower efficiency [50,51]. This enzyme is frequently found in fungi, yeast and plants; however, it is absent in animals and seldom present in prokaryotes [48]. Although very rare, some bacterial PDCs have been described and even produced recombinantly [49,52,53].

Generation of an acetaldehyde is an essential step in the fermentative production of ethanol, followed by the conversion of acetaldehyde to ethanol mediated by alcohol dehydrogenases [48,50,54,55]. This step is considered to be the primary elimination step for pyruvate in the production of lactic acid by *Rhyzopus oryzae* [48], while both lactate dehydrogenases and PDC share the same substrate. It is not surprising that PDC is involved in the regulation of ethanol production. In plants, it plays a role in pH regulation in anaerobiosis and respiration support [48] and is involved in aroma formation [51].

A recent study by Wang et al. (2019) showed that one PDC of melon could decarboxylate 2-oxobutanoate, 2-oxopentanoate and 2-oxohexanoate, forming propanal, butanal and pentanal, respectively, as well as branched α-ketoacid chains. This study was carried out to determine whether the enzyme was involved in aroma formation, since it is highly active in ripe fruit. The kinetic properties of melon PDC1 toward accepted substrates were also established [51]. These findings could be very useful in the design of new biocatalytic processes for the production of food aroma compounds [9].

**Figure 7:**
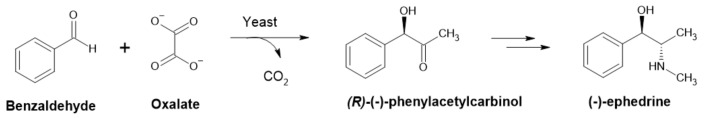
Synthesis of *(R)*-PAC and structure of (−)-ephedrine.

PDC is well known for catalyzing the biotransformation of benzaldehyde into *(R)*-phenylacetylcarbinol (*(R)*-PAC), an intermediate in the pharmaceutical synthesis of ephedrine and pseudoephedrine (Figure 7) [48,56] and drugs with anti-asthmatic and decongestant properties [57]. Using *Saccharomyces cerevisiae* and glucose and benzaldehyde as substrates, this was the first commercialized chiral biocatalysis [56]. Its *(S)*-configured counterpart (*(S)*-PAC) is used for the synthesis of phenylpropanolamine-type drugs such as cathine [53]. An engineered strain of *E. coli* with reduced aromatic aldehyde reduction (RARE) was used for synthesis of *(R)*-PAC from exogenously added benzaldehyde and pyruvate from glucose metabolism with less than 4% reduction of benzaldehyde to benzylalcohol [58].

#### 2.2.4. Acetohydroxyacid Synthase

Acetohydroxyacid synthases’ (AHAS) physiological function is the conversion of two molecules of pyruvate to acetolactate, while releasing CO_2_. It is the first step in biosynthesises of branched-chain amino acids [59]. However, it exhibits activity for PDC to form acetaldehyde [55,56]. PDC activity of AHAS was first reported in 2016 by Eram and Ma on AHAS from the hyperthermophilic bacterium *Thermotoga maritima* (conversion of 2-ketoisovalerate to isobutyraldehyde, with a rate approximately 10% of that when pyruvate was used as substrate) [55]. While the study aimed to answer questions about the source of aldehydes in the metabolism of ethanol in extreme thermophiles, its results triggered extensive research of this enzyme regarding its ability to catalyze the formation of *(R)*-PAC with an enantiomeric excess over 98% [56,57].

#### 2.2.5. α-Ketoisovalerate Decarboxylase

**Figure 8:**
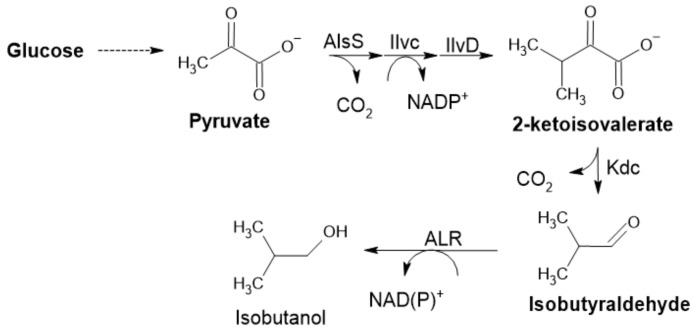
Isobutyraldehyde pathway in *E. coli*, with the aldehyde-forming enzyme 2-KID (Adapted with permission from ref. [7]. Copyright 2014 Elsevier).

Another enzyme included in the pyruvate decarboxylase group of enzymes is α-ketoisovalerate decarboxylase (KID). It is involved in the catabolism of amino acids, acting on α-keto acids formed by initial transamination. This reaction is driven by the irreversible formation of CO_2_, the same principle in the case of PDC (Figure 6) [1,60]. KID was applied not only for synthetic (in vitro or in vivo) purposes but also for food aroma development; mainly in cheese, where it is essential for flavor development control (possibly creating new cheese flavors) [60]. It plays an important role in isobutanol production by *Clostridium cellulolyticum* (Figure 8). This KID was recently used for improving isobutanol production in *Synechocystis* PCC 6803. Rational design was used to engineer the substrate binding pocket to increase catalytic activity [61].

## 3. Aldehydes Produced by Enzymatic Cascades

The current interest in multi-enzymatic processes with no need for the separation of intermediates is well established approach for synthesis of valuable chemicals with many various applications. The strategy of coupling more enzymes in vitro or in vivo cascade helps to design sustainable processes with low impact on the environment [62], while providing an opportunity to keep concentrations of potentially toxic intermediates to a minimum [13,36,63,64].

**Figure 9:**
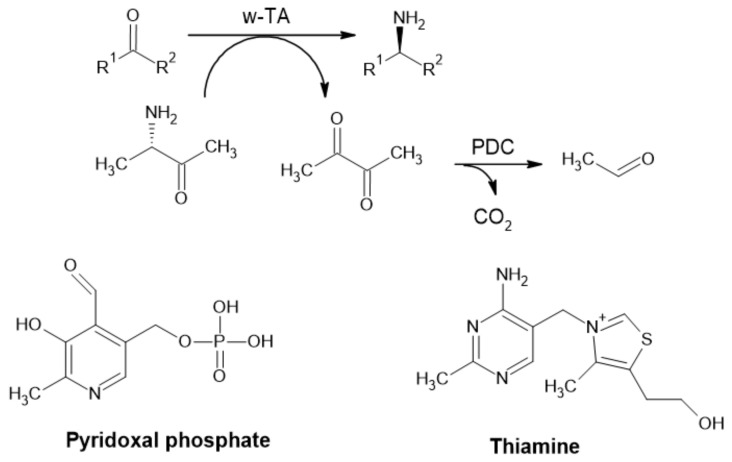
Synthesis of an α-chiral primary amine from ketone, employing ω-transaminase (ω-TA) and removal of pyruvate by pyruvate decarboxylase (PDC) (Adapted with permission from ref. [4]. Copyright 2011 Wiley) and cofactors of ω-TA.

The high reactivity of aldehydes designates them to serve mostly as intermediates or as precursors (e.g., pharmaceuticals) [1]. Furthermore, in PDC applications, pyruvate is degraded to a highly volatile acetaldehyde and CO_2_, assuring irreversibility and can be used for shifting reaction equilibria (Figure 9) [4].

### Lipoxygenase Pathway

The lipoxygenase pathway is the source of C_6_ and C_9_ aldehydes, chemicals with interesting organoleptic features widely used in perfumery and the food industry. It involves two enzymes: lipoxygenase (LOX) and hydroperoxide lyase (HPL) [65]. It is a part of the oxylipin methabolic pathway in plants, one of the most important pathways through which herbivory attack and bacterial or fungal infection defense-related genes are involved and a variety of secondary metabolites are produced. By oxidation of polyunsaturated fatty acids, plant oxylipins are produced: e.g., jasmonates, divinylethers and green leaf volatiles (GLVs) [66]. This pathway is interesting not only for its possible application to synthesize aldehydes (biotransformation of oil hydrolysates) but also for its natural occurrence in food [67].

Lipoxygenase (LOX, linoleate:oxygen oxidoreductase, EC 1.13.11.12) is a dioxygenase. It catalyzes the oxidation of polyunsaturated fatty acids and lipids containing a *cis, cis*-1,4-pentadiene structure [68,69]. The main natural substrates are linoleic and linolenic acid that are converted to 9- or 13- fatty-acid hydroperoxides depending on the source of the enzyme and reaction conditions. Hydroperoxides are substrates for the corresponding 9- or 13-hydroperoxide lyase [65,70,71].

**Figure 10:**
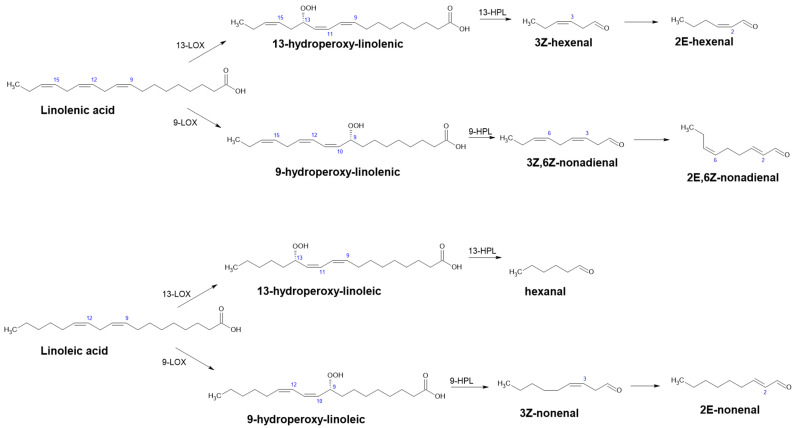
Possible products of the lipoxygenase pathway and alternative products after enzymatic reduction (Adapted with permission from ref. [72]. Copyright 2016 Academic Press).

Hydroperoxide lyase (HPL) catalyzes homolytic isomerization of fatty-acid hydroperoxides to unstable hemiacetals, which decompose to produce an aldehyde and oxo-acid (Figure 10). In the case of linolenic acid (18:3) as a starting substrate, the cascade gives (3Z)-hexenal and 12-oxo-(9Z)-dodecenoic acid for 13-HPL and (3,Z,6,Z)-nonadienal and 9-oxo-nonanoic acid for 9-HPL. Since linoleic acid (18:2) lacks one double bond at C_15_ compared with linolenic acid (18:3), only the cleaved aldehyde is affected. In this case, the cascade produces hexenal (13-HPL) and (Z)-3-nonenal (9-HPL) [67,70,72]. Apart from the aforementioned aldehydes, GLVs consist of their respective alcohols and esters and are released under stress conditions. Notably, they comprise many components in the blend of volatiles involved in interplant communication. Their importance in plant pathogen response has been recognized and, thus, they have been studied since the early 1990s [66].

Despite being known as “hydroperoxide lyase” since 1976 or as the “hydroperoxide cleavage enzyme system” before that [73], HPL has not been registered by the Enzyme Commission (EC) on account of incomplete knowledge of its reaction mechanism. In 2018, Mukhtarova et al. provided evidence of short-living hemiacetals, suggesting replacement of this name with “hemiacetal synthase”.

Even though many studies showed laboratory applications of this pathway for the synthesis of GLVs, the industrial applications (large scale-conversion of hydroperoxides to aldehydes) are limited due to the instability of HPL. It has an affinity for membranes and destabilizes quickly when extracted with detergent. HPL also displays a suicide-like mechanism and its catalytic activity is also irreversibly inhibited by products of the reaction [74]. This inactivation is a common feature of HPLs and other enzymes of the P450 family and has been linked to destruction of the prosthetic group (heme) [70].

One of the solutions that may overcome this phenomenon is HPL immobilization. An acidified reaction mixture of 13-HPOD (hydroperoxyoctadecadienoic acid) synthesis (by lipoxygenase from soybean type I-B) was used for an HPL reaction. HPL was sourced from leaves of *Amaranthus mangostanus*, immobilized on chitosan hydrogel particles, and used in a packed bed reactor for continual synthesis of hexanal. This system has a high application potential, while achieving the largest volumetric productivity: 3.560 ± 0.130 g L^−1^ when 16 U of immobilized HPL was used to catalyze 0.010 L 0.04354 M 13-HPOD at the residence time of 61 min. This is due to the high operational stability of HPL that resulted from immobilization and a higher ratio between HPL conversion and hexanal, which exhibited a lower inhibition effect than 13-HPOD [74].

Despite being a source of many valuable chemicals, this pathway is still exploited mainly for biocatalytical purposes [75] and the engineering of microorganisms with combined enzymes to produce GLVs de novo has not yet been reported. The overexpression of various LOX and HPL genes in *E. coli, Saccharomyces cerevisiae*, or plant *Nicotiana benthamiana* and designing efficient biosynthetic strategies show promising future applications of this process. Modifications in plant signaling pathways could be used to control plant switches. This strategy can be used not only to generate various bioactive chemicals using microorganisms and plants but also to enable them to produce their own pesticides. This could protect plants and eliminate the use of toxic herbicides and antimicrobials for food production [66].

## 4. Engineering of Whole Cell Biocatalysts

There are two main approaches in the production of aldehydes. First, the engineering of a microorganism that can express certain enzymes or metabolic pathways, used as a whole-cell biocatalyst or source of an enzyme. Second, the development of microorganisms that are also capable of accumulating aldehydes. While most aforementioned reactions can be accomplished by the application of one or more enzymes in vitro, only a few technologies for the bioaccumulation of aldehydes in vivo have been reported. Even though many enzymatic routes for aldehyde production are used, aldehyde toxicity still prevents higher accumulation. Recent strategies to bypass this problem include in situ separation of aldehydes (stripping), two-phase systems or selective resins. These methods can be used independently of the mechanism of aldehyde toxicity and are useful until the precise mechanisms of aldehyde’s impact on cells are clarified. Some bacteria evolved to form microcompartments that feature aldehydes, in contrast to the standard solution of their rapid reduction [1]. However, their production as powerful, highly reactive intermediates has been useful in the synthesis and bioaccumulation of other valuable chemicals, such as various unlikely alcohols, alkanes and esters.

In recent years, progress in the production of various enzymes and the creation of new biocatalysts has been made. Better operational stability under novel conditions and high productivity are expected features. To prevent changes in conformation and, thus, changes in the catalytic activity of enzymes in non-conventional conditions, whole-cell biocatalysts are usually applied. This is because microbial cells still provide the best-known environment for enzymes. Advances in design, development, engineering and applications of whole-cell biocatalysts were covered by many reviews [11,76,77,78,79,80,81].

### 4.1. Engineering of Microorganisms for the Accumulation of Aldehydes

Another approach to aldehyde synthesis/production is the use of microorganisms engineered for the bioaccumulation of aldehydes. This approach was covered in a mini review [1] and summarizes the progress from chemical synthesis to bioaccumulation and distinguishes between two approaches, constructing microorganisms to generate aldehydes (to act as a biocatalyst) or to accumulate aldehydes. This approach usually needs metabolically active cells to supply and/or regenerate cofactors [1], while only some applications/approaches demand actively growing cells.

There are two main reasons to engineer microorganisms in the context of aldehyde synthesis: to produce the desired aldehyde; or to produce a specific aldehyde that is subsequently converted to a desired product, such as an alcohol or alkane [7]. While the second reason can indicate that the aldehyde, as an intermediate, is not at the center of interest, the opposite is true. Subsequent reactions, the conversion to an alcohol (aldehyde reductase (AHR)), alcohol dehydrogenase (ADH), or alkane by aldehyde decarbonylase, are quite simple to carry out. Moreover, conversion to alcohols seems to be carried out by uncharacterized enzymes that exhibit promiscuous ADH activity, e.g., in *E. coli* [82]. In 2014, Kunjapur et al. reported the construction of a RARE *E. coli* strain based on *E. coli* K-12 MG1655(DE3) for aromatic aldehyde synthesis. The engineered strain bares seven deletions: three genes encoding aldo-keto reductases (AKRs), three ADHs and *yqhC*, an activator of the transcription of AKR with the greatest reported activity towards benzaldehyde) [58]. Rodriguez and Atsumi reported the deletion of 13 genes in multiple *E. coli* strains. Deletion of these genes led to a major elimination of ethanol as a main by-product of the conversion of pyruvate to acetaldehyde; there was 99.8% less ethanol produced in pyruvate supplemented media after deletions in *E. coli* JCL260 harboring pSA129. These combined deletions led to a 90–99% reduction of *E. coli* endogenous aldehyde reductase (ALR) activity toward various C_2_−C_12_ aldehydes. This engineered *E. coli* strain could be further used in aldehyde synthesis for in vivo screening of ALR activity by *E. coli* expressing the isobutyraldehyde pathway (Figure 8) [7]. It is clear that this field is very progressive, however, there are still no reports on industrial or upscaled processes [40].

### 4.2. Recombinant Biocatalysts

Molecular cloning, expression in a suitable host and, subsequently, characterization have become standard procedures for enzymatic studies. Genetic constructs used for these purposes frequently use synthetic DNA, mostly due to genomic and metagenomic sequencing projects. Heterologous expression is the key for successful enzyme production and is usually linked with optimized codon usage genes. However, favored codons are not those that occur most frequently in highly expressed *E. coli* but those read by tRNAs that are mostly charged when starving on amino acids [83].

Even after optimization of a gene and its expression in a suitable host, it is not guaranteed that an active protein will be produced. Expression in *E. coli* is a relatively easy and fast way to synthesize large amounts of protein. However, *E. coli* as a host is mostly not able to provide sufficient cofactors or post-translational modifications, or fold the enzyme properly, resulting in inactive protein formation.

#### 4.2.1. Biosynthesis and Regeneration of Cofactors

##### Heme Biosynthesis for HPL Expression

The first example of bottleneck in cofactor synthesis is an expression of hydroperoxide lyase (HPL) in *E. coli*. In this case, the host heme biosynthetic pathways rate-limiting step is the formation of δ-aminolevulinic acid (δ-ALA) [84]. A strategy to overcome this limitation is to feed δ-ALA directly to the cultivation media [70,85]. Interestingly, when producing HPL sourced from guava fruit (*Psidium gujava*) in *E. coli* BL21(DE3), the best results, in terms of activity, were obtained without δ-ALA supplementation and even without the addition of an inductor, isopropyl-β-D-thiogalactopyranoside (IPTG) [85]. However, Sudhamsu et al. (2010) showed that the insertion of ferrous iron into protoporphyrin IX became the rate-limiting step under conditions of supplementation with δ-ALA.

Indirect evidence of improved incorporation of the heme prosthetic group into recombinant HPL (also sourced from *Psidium gujava*) after directed evolution has been reported. A four-fold increase in heme concentration in cells expressing an improved variant of the gene was observed. Authors estimated 67% heme incorporation in the final variant compared with 15% in the parental protein (HPL from *Psidium gujava* fused with maltose binding protein MBP). In this case, directed evolution increased the product yields. Interestingly, most sequence alternations of the best variant enzymes happened on the surface, outside of well-defined secondary structures, and none of the altered amino acids were involved in the active center of those interacting with the prosthetic group [70].

##### Post-Translational Modifications of 4-Phosphopantetheine (Ppant)

Due to the post-translational modifications of CARs in *E. coli*, expression is problematic. Incubation of purified recombinant CARs with cell-free extracts of *Nocardia* CFE and coenzyme A (CoA) resulted in a five-fold specific activity increase [19]. In vitro post-translational activation of CAR from *Mycobacterium marinum* with *Geobacillus kaustophilus* PPTase in the presence of CoA resulted in a 10% higher yield of piperonal from piperonylic acid [86].

Within in vivo interventions, co-expression of CAR and PPTase of *Nocardia* in *E. coli* reached a 20-fold higher specific activity [19]. Various PPTases were tested for this purpose (PPTase from *E. coli*, *Nocardia iowensis* and *Neurospora crassa*), but best results regarding to activity were achieved by *Mycobacterium marinum* and *Bacillus subtillis* genes. However, a higher activity of PPTase does not imply an increased activation of apo-CAR, which suggests that the interaction of enzymes is more important than their concentration. Equimolar concentration of intracellular CoA to apo-CAR is needed for full modification by PPTase. The biosynthesis of CoA can be supported by the supplementation of growth media with its precursors so its depletion is avoided (addition of 0.005 M of D-pantothenate prior to expression increased CAR activity by 35%) [86].

Before the application of the obtained active enzyme in in vitro biotransformations, the removal or regeneration of the by-product’s reaction must be addressed. This can be achieved by the integration of other enzymes in vitro [21] or using a whole-cell biocatalyst.

##### NAD(P)H Regeneration

A lot of industrially important oxidoreductases require stoichiometric amounts of cofactors for their catalytic function. The low stability and high price of NAD(P)H caused development of various systems for their regeneration [87]. Traditional approaches include electro-enzymatic reactions, the combination of enzymes for cofactor regeneration (e.g., glucose dehydrogenase, formate dehydrogenase) or whole-cell biocatalysis [88].

An enzymatic partner with high V_max_ for the regeneration of cofactors in vitro is usually sufficient. However, boosting in vivo regeneration capacity required more delicate approach. This led to the generation of cofactor-auxotrophic *E. coli* strains serving as “biosensors” for the capability of enzymes to regenerate NADH [89] and NADPH [90] in vivo. These can be used to test and compare the ability of different enzymes to regenerate cofactors, for example, from glucose, formate or methanol as auxiliary substrates. The regeneration of NAD(P)H is important not only for *E. coli* but is studied in other industrially important microorganisms, such as *Pichia pastoris* [88], *Klebsiella oxytoca* [91] or *Bacillus subtillis* [92].

##### ATP Regeneration

Unlike well-established NAD(P) recycling systems, ATP recycling is relatively new and not routinely used yet. Therefore, systems that attempt simultaneous reuse of ATP and NADPH are quite complex and require the balanced use of compatible enzymes. CARs cofactors were regenerated by the action of polyphosphate kinase to form *Meiothermus ruber* and *Sinorhizobium meliloti* (regeneration of adenosine 5-triphosphate) and GDH (regeneration of β-nicotinamide adenine dinucleotide 2-phosphate) which resulted in the full conversion of carboxylic acids to aldehydes in cell-free system [93]. This approach was later applied as an external ATP/NADPH recycling system for whole-cell biocatalysis with CARs [86].

## 5. Analytics of Aldehydes Used in Biocatalysis

### 5.1. Photometric Methods

Since aldehydes are highly reactive molecules, there are only a few chemical agents for their spectrophotometric detection. This makes high throughput screening by the direct measurement of aldehyde products rather hard to achieve, and only a few reliable methods exist. Instead, the quantification of other chemicals involved is used, such as a decrease in substrate, cofactor transformation, or stoichiometric by-products. This list is not supposed to be exhaustive, but it illustrates the methods used for screening the activity of aldehyde-producing enzymes.

#### 5.1.1. Detection of Aldehyde Products

##### Luciferase Assay

**Figure 11:**
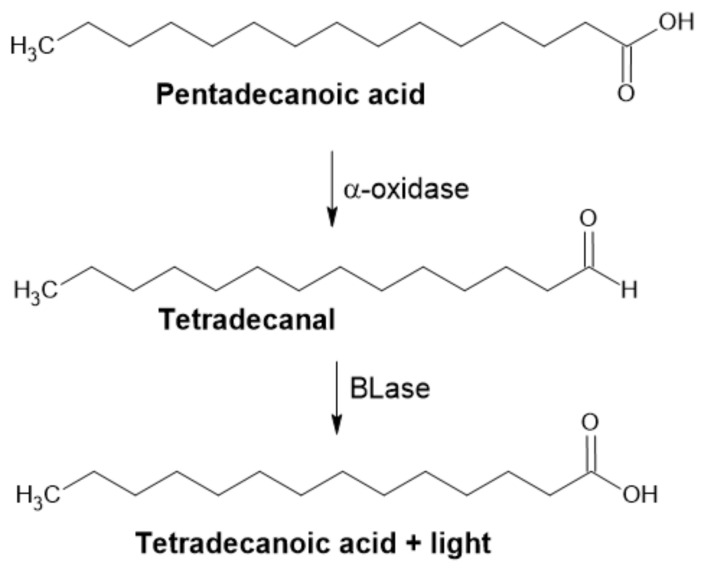
Application of BLase for detection of α-DOX activity.

The luciferase assay was first reported in 2000 by Cho. This author demonstrated the use of bacterial luciferase (BLase) for oxidizing long-chained fatty aldehydes to corresponding fatty acids. In the presence of molecular oxygen and reduced flavin, a mononucleotide emission of blue-green light (λmax ≈ 490 nm) accompanies the reaction (Figure 11). This method is based on the measurement of emitted light and has very few background signals, which contributes to its high sensitivity and reproducibility. This simple assay was used in microtiter plates (96-well) in a total volume of 0.2 × 10^−3^ L for the detection of α-DOX activity [43].

##### Detection of Formaldehyde by Acetylacetone and Ammonium Salt

**Figure 12:**
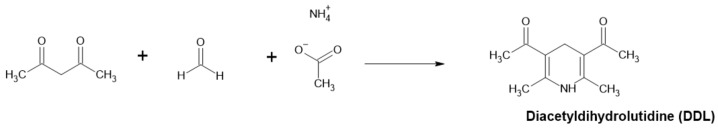
Detection of aldehyde by acetylacetone and ammonium salt.

After AOX catalyzed the oxidation reaction of methanol to formaldehyde and hydrogen peroxide in the presence of ammonium salt, acetylacetone was added (Figure 12). This reaction yields diacetyldihydrolutidine, a yellow-colored product that can be spectrophotometrically determined at 412 nm [31].

##### Acetylacetanilide Method

**Figure 13:**
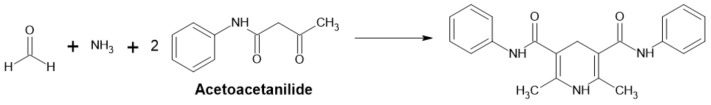
Detection of aldehyde by acetylacetanilide.

The concentration of formaldehyde can also be measured by the acetylacetanilide reagent (25 × 10^−6^ L 0.59 M acetylacetanilide reagent in DMSO/water, 80/20 *v/v*). The product can be determined by measuring the intensity of fluorescent light at 460 nm (excitation wavelength 360 nm) (Figure 13) [31].

##### Basic Fuchsin Method

**Figure 14:**
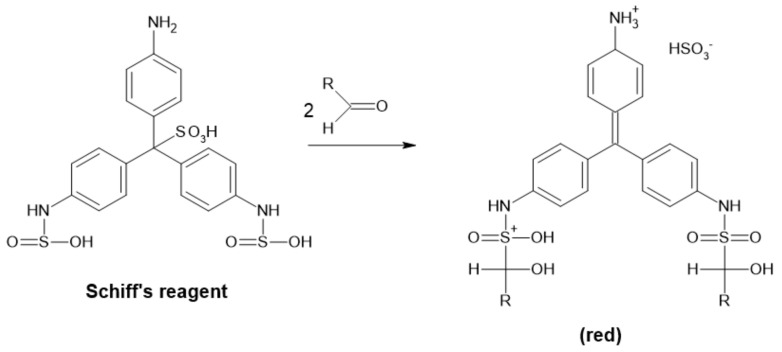
Detection of aldehyde by Schiff’s reagent.

Formaldehyde can also be measured by a reaction with the basic fuchsin reagent (0.1% basic fuchsin in 1% Na_2_SO_3_ and 1% H_2_SO_4_ solution). In this case, the product of the reaction is violet-purple and can be determined at 560 nm (Figure 14) [94].

##### Amino Benzamidoxime Assay

**Figure 15:**
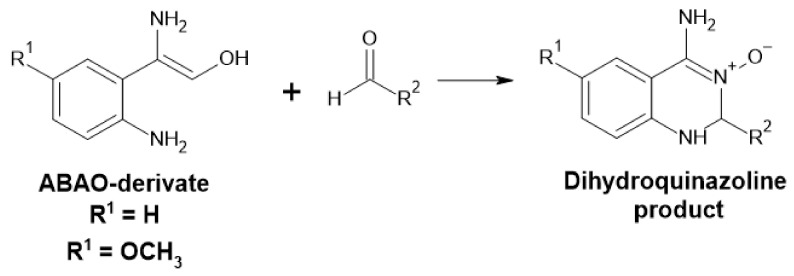
Detection of aldehyde by ABAO derivates.

Measurement of the concentration of various aldehydes can be accomplished by the addition of the 2-amino benzamidoxime derivates (ABAO) assay (Figure 15). The resulting dihydroquinazoline product can be used for quantification at 380 [95] or 405 nm [96] in µM range and semi-quantification at nM range using fluorescence. This method can be used for high-throughput screening and is chemo-selective for aldehydes [95,96].

#### 5.1.2. Detection of Other Involved Chemicals

##### ABTS-POD Method

**Figure 16:**
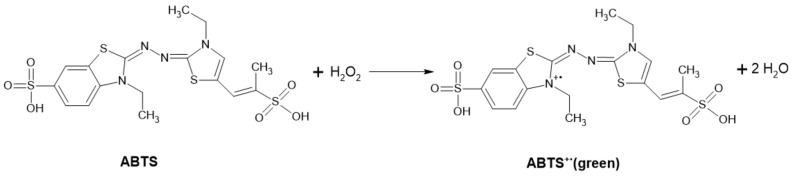
Scheme of the ABTS peroxidase coupled reaction.

This method is based on the measurement of the stoichiometric amount of hydrogen peroxide produced as a by-product of some enzyme reactions. Oxidation of ABTS^TM^ (2,2′-azino-bis-(3-ethylbenzothiazoline-6-sulfonic acid)) is spectrophotometrically recorded at 405 nm [29]. The arising green coloration is due to the formation of a stable green ABTS^+^ radical (Figure 16) [97]. Measurement at multiple wavelengths, including at 405 [29], 415, 650, 732 and 820 nm, is possible with different sensitivities [97]. 

##### Disruption of the Conjugated Double Bond System—HPL Reaction

While C_6_-aldehydes produced from the lipoxygenase pathway exhibit low optical activity, it is hard to establish a high-throughput screening activity assay for HPL products. Instead, disruption of the conjugated double bond system is detected with a decrease in absorbance at 234 nm. In this case, measurement of the substrate decrease is carried out instead of measuring the product concentration. This method does not differentiate different hydroperoxides (for example, a mixture of 9- and 13-hydroperoxides after synthesis with LOX), therefore, the absolute concentration of a substrate (for 9- or 13-HPL) must be checked by other means [70]. Although it has been used to measure the activity of HPL in various conditions, it is often replaced by gas chromatography methods [70,98].

##### NAD(P)H Spectrophotometric Assay

This simple spectrophotometric assay is based on the difference in the absorption spectra of oxidized and reduced nicotinamide cofactors of various enzymes. It is very well known and widely used. The reduced form of NAD(P)^+^ exhibits an absorption maximum at 340 nm and can be measured to monitor the reaction. The stoichiometry of catalyzed reactions is known, and its rate can be easily calculated. This convenient method can be used to monitor the activity of CARs and is reliable even in HTP screenings [16,19,21].

### 5.2. Chromatographic Methods

Aldehydes are very important industrial chemicals and, therefore, there are many chromatographic methods for their quantification. Combining them with the use of various derivatization techniques can make analytes more susceptible to detection and quantification in general. This is particularly important in chromatographic techniques, where it does not only improve resolution and symmetry but can also produce more stable (chemically or thermally) analytes and, thus, improve separation [10].

While biological samples, including food samples, can be very complex matrices, consisting of many different compounds, ideal preparation of the sample should not only protect the chromatographic column but simultaneously make detection possible.

High volatility makes aldehydes ideal candidates for gas chromatography analysis, with an FID detector being one of the most frequently used. Extraction of the reaction mixture (or culture) is a common practice for sample preparation [7,70]. Another approach is the use of a headspace sampler and heating sample to reach equilibrium between the gas and liquid phases. The gas phase is then subsequently analyzed [74]. This greatly lessens the amount of analyte lost due to evaporation, mainly when higher operational temperatures are used.

Quite often, the methods for modification of reactive aldehydes are applied. Products of 13-HPL are reduced by NaBH_4_ to convert C_6_-aldehydes to their corresponding alcohols and are subsequently extracted and analyzed by GC with an FID detector [70].

**Figure 17:**
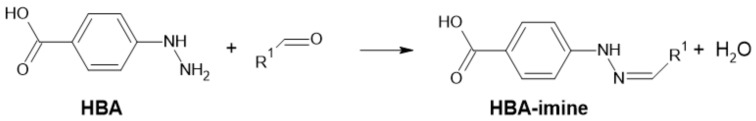
Derivatization reaction of 4-HBA and aldehyde (Adapted with permission from ref. [10]. Copyright 2018 Elsevier).

One of the derivatizing agents for the analysis of aldehydes is 4-hydrazinobenzoic acid (4-HBA). It can undergo nucleophilic addition to aldehydes or ketones using hydrazones, which can be detected at 320 nm after separation by HPLC (Figure 17).

However, this is not the only way to make carbonyl compound analysis possible. Many other derivatizing agents exist with their own advantages and disadvantages. The complexity of natural matrices makes developing new analytical processes for aldehydes complicated. By integrating new technology and advanced analytical methods, optimal sample preparation and analytical processes can be developed. Combining 4-HBA derivatization with gas-diffusion microextraction allowed for the determination of low molecular weight aldehydes, with only one derivate forming from each aldehyde. This method was developed to be used for complex samples since it uses simultaneous sample concentration and clean-up [10].

### 5.3. Biosenors

Even though aldehydes can be quantified by the methods mentioned above, analytical throughput of these techniques is limited. Development of screening and selection methods are vital for the further exploitation of directed evolution and other methods based on random mutagenesis. Metabolite sensors are central to the interest in evolution of new enzymes and bacterial strains [99].

#### 5.3.1. Ligand-Responsive Transcription Factors

Ligand-responsive transcription factors are DNA-binding proteins that regulate gene expression by interacting with specific molecules. They can be used for the construction of biosensors comprised of a sensing and reporter module. The sensing module contains the transcriptional factor, activated by the target ligand, and the reporter module consists of a corresponding promoter that regulates the transcription of the reporter gene that produces a measurable signal. A YqhC transcriptional regulator was used for high-throughput detection of aldehydes in *E. coli* [99]. A vanillate biosensor based on vanillate-responsive transcriptional repressors can be used for engineering industrially relevant de novo vanilline biosynthesis [100].

#### 5.3.2. Luciferase-Based Biosensors

The specificity of BLase for long-chained aldehydes can be exploited for monitoring aldehyde production in real-time during cell growth. This method can be used for the measurement of the intracellular concentration of aldehyde intermediates, for example in the production of wax esters [101,102] or alkanes [103].

## 6. Products/Applications

The diversity of aldehyde applications brings specific requirements for its products, which are based on product application. In the cosmetic industry, consumers tend to prefer products that can be labelled as “natural” and, therefore, many microbial and enzymatic methodologies are being used [9]. Exhibiting interesting organoleptic features, aldehydes could be in the position of unwanted odors. This is why the study of aldehyde-producing enzymes or enzyme cascades is not limited to their synthesis but is also focused on a better understanding of exploiting or enhancing naturally occurring routes for flavor enhancement [67,104]. Studying natural processes brings new opportunities in biosynthesis, not only for fine chemicals but also for complex flavor formation; for example, studying the ripening of melon and formation of acetaldehyde, propanal and pentanal using pyruvate/α-ketoacid decarboxylase [51]. In this field, it is mostly the naturally occurring pathways that are being studied, and production is mediated by nature-sourced enzymes (e.g., plant homogenates from various sources) [72,74]. An interesting exception is vanillin (4-hydroxy-3-methoxybenzaldehyde), which is widely used for its distinctive flavor and smell. Chemically synthetized vanillin accounted for 99% of the total market share [9] and it is now the second most demanded flavor (after saffron). Biotechnological production of vanillin was summarized in a recent review [8].

## 7. Conclusions

Aldehydes, a diverse class of highly reactive chemicals, have been at the center of academic and industrial interest for many years. While they are mostly known for their distinctive organoleptic features, they are useful in many sectors of industry, including pharmaceutical, plastic production and (bio)fuel applications. Other specific features, mainly high reactivity and the resulting toxicity, make production of aldehydes challenging and, thus, technology development requires an individual approach.

The application of biotechnological approaches, use of advanced techniques for genetic and metabolic engineering and integration of effective computational methods all contributed to the rise of novel technologies. Synthetic biology helped to establish “greener” technologies for the production of both bulk and fine chemicals. In contrast, the high reactivity and instability of aldehydes predetermines them to be used as short-living intermediates. Aldehyde-producing enzymes or enzyme cascades occur in raw food materials and, thus, control of their activity is a top priority. In food and aroma industries, demand for “natural” products is predominant. These specific requirements, from evolving odors (e.g., in cheese) to synthesis of natural food additives, gave rise to technologies exploiting various enzymes.

De novo aldehyde synthesis and their accumulation in microorganisms comprise another branch in the biotechnology of aldehydes. No wild-type microorganism is known to accumulate aldehydes. With the introduction of genetic tools, microorganisms can produce and accumulate them to some extent. These new technologies possess great potential for the future production of aldehydes.

## Abbreviations

(h)DAO	(human) Diamine oxidase
13-HPOD	hydroperoxyoctadecadienoic acid
4-HBA	4-hydrazinobenzoic acid
ABTS	2,2′-azino-bis-(3-ethylbenzothiazoline-6-sulfonic acid)
ADH	Alcohol dehydrogenases
AHAS	Acetohydroxyacid synthases
ALR	aldehyde reductase
AMP	Adenosine monophosphate
AOs	amine oxidases
AOX	Alcohol oxidase
ATP	Adenosine triphosphate
BLase	bacterial luciferase
CAR	Carboxylic acid reductase
CoA	coenzyme A
DOX	Dioxygenase
FAD	flavine adenine dinucleotide
GLVs	green leaf volatiles
HPL	hydroperoxide lyase
IPTG	Isopropyl-β-D-thiogalactopyranoside
KID	Ketoisovalerate decarboxylase
LOX	Lipoxygenase
NAD(P)H	Nicotinamide adenine dinucleotide (phosphate)
PCD	Peptidyl carrier domain
PDC	Pyruvate decarboxylase
Ppant	4-phosphopantetheine
PPTase	Phosphopantetheine transferase
*(R)*-PAC	R-phenylacetylcarbinol
TAT	Twin-arginine translocation
ThPP	Thiamine pyrophosphate
δ-ALA	δ-aminolevulinic acid
